# The effect of electrical stimulation on corticospinal excitability is dependent on application duration: a same subject pre-post test design

**DOI:** 10.1186/1743-0003-10-51

**Published:** 2013-06-10

**Authors:** Rebecca K Andrews, Siobhan M Schabrun, Michael C Ridding, Mary P Galea, Paul W Hodges, Lucinda S Chipchase

**Affiliations:** 1 School of Health and Rehabilitation Sciences and the NHMRC Centre of Clinical Research Excellence in Spinal Pain, Injury and Health, The University of Queensland, St Lucia, Brisbane, Queensland 4072, Australia; 2 The Robinson Institute, School of Paediatrics and Reproductive Health, The University of Adelaide, Adelaide, South Australia 5005, Australia; 3 Rehabilitation Science Research Centre, The University of Melbourne, Melbourne, Victoria 3010, Australia; 4 School of Science and Health, University of Western Sydney, Campbelltown, NSW, Australia

**Keywords:** Electrical stimulation therapy, Homeostatic plasticity, Transcranial magnetic stimulation, Physical therapy modalities, Rehabilitation

## Abstract

**Background:**

In humans, corticospinal excitability is known to increase following motor electrical stimulation (ES) designed to mimic a voluntary contraction. However, whether the effect is equivalent with different application durations and whether similar effects are apparent for short and long applications is unknown. The aim of this study was to investigate whether the duration of peripheral motor ES influenced its effect on corticospinal excitability.

**Methods:**

The excitability of the corticomotor pathway to abductor pollicis brevis (APB) was measured in fourteen health subjects using transcranial magnetic stimulation before, immediately after and 10 minutes after three different durations (20-, 40-, 60-min) of motor ES (30Hz, ramped). This intervention was designed to mimic a voluntary contraction in APB. To control for effects of motor ES on the peripheral elements (muscle fibre, membrane, neuromuscular junction), maximum compound muscle actions potentials (M-waves) were also recorded at each time point. Results were analysed using a repeated measures analysis of variance.

**Results:**

Peripheral excitability was reduced following all three motor ES interventions. Conversely, corticospinal excitability was increased immediately following 20- and 40-min applications of motor ES and this increase was maintained at least 20-min following the intervention. A 60-min application of motor ES did not alter corticospinal excitability.

**Conclusions:**

A 20-min application of motor ES that is designed to mimic voluntary muscle contraction is as effective as that applied for 40-min when the aim of the intervention is to increase corticospinal excitability. Longer motor ES durations of 60-min do not influence corticospinal excitability, possibly as a result of homeostatic plasticity mechanisms.

## Background

Electrical stimulation (ES) is widely used in the rehabilitation of many neurological conditions, particularly those that involve damage to the central nervous system [1-7]. When the aim is to enable motor function, ES can replace or assist a patient’s voluntary attempts to execute or control a functional movement [8]. Motor ES, or functional electrical stimulation as it is otherwise known, has been demonstrated to improve function in individuals with stroke, multiple sclerosis and spinal cord injury [1-7].

Peripheral mechanisms underlying improved function following motor ES have been extensively examined and changes in muscle structure and function have been confirmed [9-14]. These changes include increased oxidative capacity of muscle, increased myocapillarisation, and transformation of muscle fibre types [9-14]. Motor ES also strengthens synaptic connections in the ventral horn leading to altered excitability of spinal motoneurons [15,16]. Although peripheral changes explain some of the improvements in strength and functional capacity gained with motor ES [1-5,7,17,18], they fail to explain changes in motor learning and skill acquisition [2,5].

Motor learning and skill acquisition are associated with increased corticospinal excitability [2,5,19-21]. Examination of corticospinal excitability in humans with transcranial magnetic stimulation (TMS) has revealed increased corticospinal excitability following motor ES [2,5,20,22-27]. The mechanisms believed to be associated with this increase in corticospinal excitability include unmasking of silent synaptic connections and long-term potentiation (LTP) of synaptic efficacy [28-31].

We have recently demonstrated that motor ES designed to mimic voluntary contractions (30 Hz stimulation with ramped stimulus amplitude), as is used in functional electrical stimulation, induced greater increases in corticospinal excitability than muscle twitches (10 Hz stimulation without ramped stimulus amplitude) [28]. Increased corticospinal excitability following motor ES (30 Hz, ramped) has been documented in healthy subjects and in those with neurological conditions [2,4,5,18,28,32].

Although the duration of ES application is likely to influence the effect of stimulation, this has not been systematically investigated. Positive effects of 1–10 Hz ES on excitability of the corticomotor pathway have been demonstrated when applied for 10-, 30-, 60- and 120- min [20,22-26,33]. However, whether the effect is equivalent with clinically meaningful parameters (e.g. 30 Hz, ramped) designed to mimic a voluntary contraction is unknown. This study aimed to investigate the effect of three clinically achievable durations (20-, 40- and 60-min) of motor ES (30 Hz, ramped) on the responsiveness of the corticomotor pathway. Based on previous research, we hypothesized that longer application times would induce the greatest change in corticospinal excitability.

## Methods

### Participants

Fourteen healthy, right-handed individuals (9 females; age 23.07 ± 7.10 years [mean ± SD]; range 18–47 years) participated in this study. Based on a minimum detectable difference in means of 0.32 mV and a standard deviation of 0.26 mV from our previous work [34], a sample size calculation using SigmaPlot Software (Systat, Chicago, USA) revealed 14 subjects would be sufficient to detect a statistically significant change (power 0.8, alpha 0.05) should one exist. All procedures were conducted in accordance with the Declaration of Helsinki and approved by the institutional Human Research Ethics Committee. All participants gave written, informed consent and completed a TMS safety questionnaire [35], prior to study commencement. Participants were excluded if they had any neurological conditions, injuries to their upper limb or contraindications to the application of TMS or peripheral electrical stimulation.

### Electromyography (EMG)

EMG recordings were made from the right abductor pollicis brevis (APB) using silver/silver chloride surface electrodes positioned in a belly-tendon montage. The skin under the electrodes was lightly abraded using Nuprep skin prep gel (Weaver and Company, Colorado, USA) and gauze, and then cleaned with an alcohol wipe. EMG signals were amplified 1000 times, filtered between 20–1000 Hz and sampled at 2000 Hz using a Micro 1401 data acquisition system (Cambridge, UK)^a^ and Signal 3 software (CED, UK)^b^.

### Transcranial Magnetic Stimulation (TMS)

TMS was used to provide a measure of the excitability of the corticospinal projection to APB. TMS was delivered using a Magstim 200 stimulator (Magstim Co. Ltd., Dyfed, UK)^c^ and a figure of eight coil (external wing diameter 9 cm). The coil was orientated over the left hemisphere and positioned at a 45° angle to the sagittal plane in order to induce current in a posterior-anterior direction. The optimal scalp site to evoke a response in APB was established and this point marked on the scalp. Stimulator intensity was then adjusted to evoke an EMG response in APB (termed a motor evoked potential; MEP) with a peak-to-peak amplitude of 1 mV at baseline. The same intensity was used to retest the excitability of the corticospinal projection to APB following the intervention. A target intensity of 1 mV was chosen as it places MEP amplitudes approximately in the middle of their stimulus–response curve, reducing the potential for ceiling or floor effects [36]. All TMS procedures adhered to the TMS checklist for methodological quality [37].

### Median nerve stimulation

Maximum compound muscle action potentials (M-waves) were recorded from the right APB to control for effects of ES on the peripheral elements (e.g. muscle fibre membrane, neuromuscular junction, motor axon etc.). A constant current stimulator (DS7A, Digitimer Ltd., Welwyn Garden City, UK, maximum current of 1A)^d^ was used to deliver a single electrical stimuli via surface electrodes positioned over the median nerve at the wrist (100 μs pulse duration). Stimulus intensity was set at 120% of that required to evoke a maximal M-wave (M_max_) in APB [38].

### Motor Electrical Stimulation (ES) intervention

A Chattanooga Intelect Advanced therapy system (OPC Health, Melbourne, Australia)^e^ was used to provide the ES intervention to the right APB muscle belly. Each subject was randomly assigned to a 20-, 40- or 60-min time condition using a simple random number generator and returned for a total of three sessions to complete each time condition. This ensured that the results would not be attributed to the repetition or the order of the task. Each session was spaced at least 72 hours apart. The intervention was delivered using a monophasic waveform with a pulse duration of 0.2 ms. Current was delivered at 30 Hz and ramped at a rate of six surges per min (4 sec on: 6 sec off) [18,34]. Stimulus intensity was increased until a contraction was obtained that abducted the thumb approximately 15° (ES intensity range 7.0 - 17.5 mA). This protocol was designed to mimic a voluntary contraction in the APB muscle without any voluntary effort from the subject [28]. As sham ES has been shown not to influence corticospinal excitability, a sham condition was not included [34].

### Experimental protocol

This study used a same subject repeated measures design. The subject was comfortably seated with the elbow flexed to approximately 90° and the arm and hand supported on a pillow in neutral wrist extension and full forearm supination. Three blocks of 12 baseline MEPs with one min rest between each block and one block of six baseline M_max_ values were recorded prior to the intervention. The motor ES (30 Hz, ramped) intervention was then applied for a duration of 20-, 40- or 60-min. To control for attention, subjects were directed to focus on the stimulation throughout the intervention and verbal reminders were provided every five min. On completion of the stimulation period, measures of MEPs and M_max_ were repeated. Four blocks of 12 MEPs (post_1_) were recorded immediately post intervention with a one min breaks between each block. One block of six M_max_ values were then recorded. 10 minutes after the intervention, an additional four blocks of 12 MEPs (post_2_) were recorded and the experiment concluded with the recording of one final block of M_max_ values. Thus, the post-intervention testing period lasted approximately 20-min.

### Data and statistical analyses

The peak to peak amplitude of MEP and M_max_ values were obtained and averaged for each time point (baseline, post_1_, post_2_). MEPs provide a measure of the excitability of the entire corticomotor pathway and thus, are influenced by excitability changes occurring at the motor cortex, motoneurone and in the periphery. M_max_ amplitudes provide a measure of excitability changes occurring within the peripheral apparatus (e.g. muscle fibre membrane, neuromuscular junction, motor axon, etc.). Thus, to account for changes occurring in the periphery as a result of motor ES, MEPs were expressed relative to M_max_ (i.e. MEP/M_max_) [28,36,39]. A two-way repeated-measures analysis of variance (ANOVA_RM_) was used to compare the effects of condition (20-, 40- or 60-min of motor ES) and time (baseline, post_1_ and post_2_) on MEP/M_max_ ratios and absolute M_max_ amplitudes. Where appropriate, post-hoc analyses were completed using the Holm-Sidak method. The level of significance was set at 5%. Group data are presented as mean ± standard deviation in text and mean ± standard error in the figures.

## Results

The stimulus intensity required to produce a 1mV MEP at baseline was 57.4 ± 12.8 in the 20 min condition, 54.9 ± 10.7 in the 40 min condition and 56.9 ± 11.2 in the 60 min condition. At baseline, there was no difference in the size of the MEPs expressed relative to M_max_ (20 min 5.1 ± 1.5%; 40 min 4.8 ± 2.1; 60 min 5.8 ± 1.6%, p all > 0.23), or in the absolute amplitude of M_max_ (20 min 22.4 ± 3.6 mV; 40 min 23.3 ± 5.7 mV; 60 min 21.5 ± 3.4, p = 0.16), between motor ES conditions.

**Figure 1:**
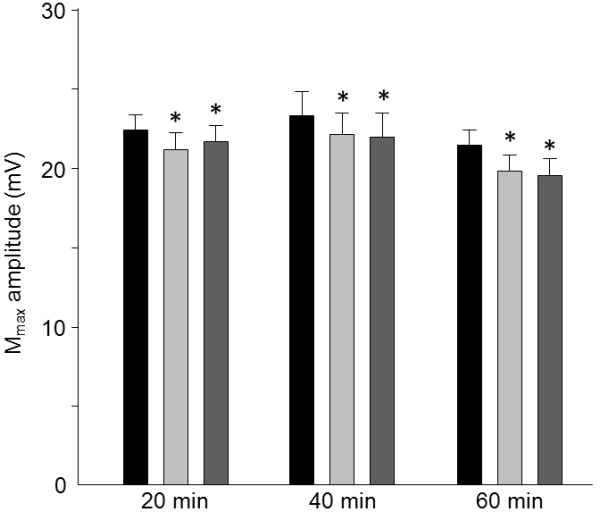
**Group data (mean ± SE) of absolute M**_**max **_**amplitudes before (black bars), immediately after (post**_**1**_**; light grey bars) and 20-min after (post**_**2**_**; dark grey bars) three motor ES durations (20-, 40- and 60-min).** M_max_ amplitude was reduced immediately after all three motor ES applications and this effect was maintained 20-min later. *p<0.05.

**Figure 2:**
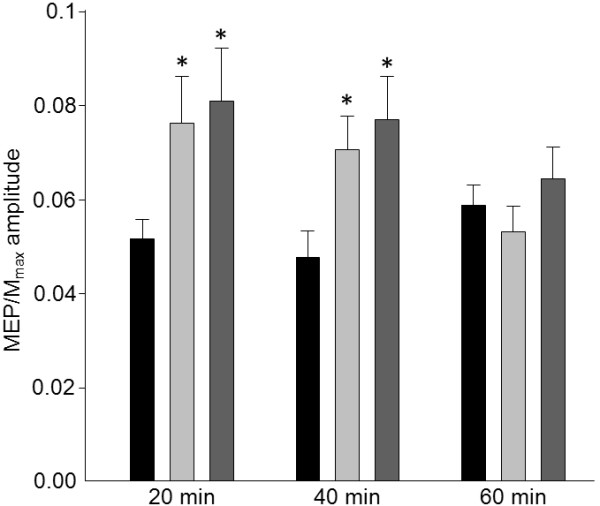
**Group data (mean±SE) of MEP/M**_**max **_**amplitude before (black bars), immediately after (post**_**1**_**; light grey bars) and 20-min after (post**_**2**_**; dark grey bars) three motor ES durations (20-, 40- and 60-min).** MEP/M_max_ amplitudes increased immediately after both the 20- and 40- min applications of motor ES and this increase was maintained 20- min later. There was no change following the 60- min application. * p<0.05.

M_max_ was reduced immediately following all three motor ES interventions (condition x time interaction, p = 0.4; main effect of time, p < 0.001; Post hoc baseline vs. post_1;_ p = <0.001), indicating that peripheral excitability was uniformly affected by ES duration. This effect was maintained at least 20-min following the intervention (Post-hoc baseline vs. post_2_; p = <0.001; Figure 1). Conversely, effects of motor ES on corticospinal excitability were affected by application time (condition x time interaction, p = 0.003; main effect of time, p < 0.001; Figure 2). Relative to M_max_, MEP amplitude increased by 48 ± 66% immediately following the 20-min (Post-hoc baseline vs. post_1_; p = 0.005), and by 48 ± 54% immediately following the 40-min (Post-hoc baseline vs. post_1_; p < 0.001), motor ES interventions. Increased corticospinal excitability persisted 20-min after stimulation in both the 20-min (57 ± 57%; Post-hoc baseline vs. post_2_; p = 0.002) and 40-min (61 ± 50%; Post hoc baseline vs. post_2_; p < 0.001) ES conditions. Excitability of the corticospinal pathway to APB did not change relative to M_max_ (post_1_ -10 ± 41% and post_2_ 9 ± 44%) when motor ES was applied for 60-min (Post hoc baseline vs. post_1_; p = 0.21; baseline vs. post_2_; p = 0.52).

## Discussion

Our data demonstrate that the effect of motor ES (30 Hz, ramped) on corticospinal excitability as measured by TMS, depends on application duration in a sample of healthy subjects. The novel findings are that although 20- and 40-min of motor ES increased corticospinal excitability, 60-min of stimulation had no effect. The similar magnitude of increase in corticospinal excitability between the 20- and 40-min conditions suggests that 20-min of motor ES is sufficient to increase corticospinal excitability.

Previous research examining the effect of motor ES on corticospinal excitability has used frequencies of 1–10 Hz and constant stimulus amplitudes to produce simple muscle twitches [20,22-26,33]. Data are conflicting with some suggesting increased corticospinal excitability with 60–120 min of stimulation [20,22,26], and others reporting increases with application times as short as 10–30 min [24,40]. The only study to systematically examine the effect of application time of motor ES (10 Hz) on corticospinal excitability reported the greatest increase in excitability with 45–60 min of stimulation [25]. As motor ES applied with a constant stimulus amplitude at 10 Hz (twitch) and that with a ramped stimulus amplitude at 30 Hz (functional) have differing effects on corticospinal excitability [28], differences in the effects of stimulation duration are possible. Recent work comparing a 30-min application of 10 Hz and ramped 30-Hz motor ES demonstrated increased corticospinal excitability only for the 30 Hz ramped protocol [28]. This suggests motor ES (30 Hz, ramped) designed to mimic a voluntary contraction can more effectively increase corticospinal excitability with short application durations, consistent with other data from short applications [2,5,18].

Similar to previous reports [32], M_max_ amplitude was reduced (indicating fatigues of the peripheral apparatus) in APB following motor ES. This effect was present regardless of ES application time. To account for these peripheral changes, MEPs were expressed relative to M_max_ in the current study. As MEPs increased with 20- and 40-min of motor ES, despite a reduction in M_max_, increased MEP amplitudes following these interventions can be attributed to excitabtility changes at the corticospinal level. However, one consideration is whether changes in corticospinal excitability following ES occur at the motor cortex or spinal motoneurones. Although not tested here, previous research has demonstrated that H-reflexes [41,42], F-waves [22] and cervicomedullary evoked potentials [19,43] are unchanged following peripheral ES. As these techniques probe motoneurone excitability, it is suggested that changes induced by ES are most likely to occur at the cortex. Several mechanisms are thought to underlie plastic change in the motor cortex following motor ES. These include unmasking of silent synaptic connections and long term potentiation (LTP) of synaptic efficacy [28-31].

Why application of ramped motor ES at 30 Hz for 60-min did not increase corticospinal excitability is unclear. One possible explanation is that time-dependent homeostatic plasticity mechanisms acted to prevent destabilisation of the nervous system and maintain neural activity within a specific range [44-47]. The long-term potentiation (LTP) and long-term depression (LTD) of synaptic efficacy, that are thought to underlie increased or decreased corticospinal excitability during ES applications, operate via a positive feedback mechanism [46]. If large increases in corticospinal excitability are induced by motor ES the potential exists for runaway excitability and destabilisation of cortical neuronal networks [48]. To ensure neural activity is maintained within a stable, physiological range homeostatic plasticity adjusts the threshold for synaptic modifications based on the history of neuronal activity [44-47]. A history of high activity biases synaptic modifications towards LTD (linked to decreased corticospinal excitability), and a history of low activity biases synapses towards LTP (linked to increased corticospinal excitability) [46,49,50]. In the current study it is possible that the first half of the 60 minute motor ES application induced an increase in corticospinal excitability sufficient to be interpreted by the system as “high activity”. This would trigger homeostatic plasticity and reduce or reverse the effect on corticospinal excitability towards that of depression. Support for this theory is drawn from a recent study by Gamboa and colleagues [50], using prolonged theta burst stimulation. Similar to our findings, a short stimulation period resulted in LTP and increased corticospinal excitability while a prolonged period of stimulation resulted in a reversal of the response towards LTD and decreased corticospinal excitability [50]. Taken together with the results of the current study, these findings suggest that longer periods of stimulation have the potential to invoke homeostatic plasticity mechanisms, reducing the effectiveness of the intervention. This novel interpretation has been overlooked in previous ES work.

Alternatively, the difference in effect of shorter and longer durations of stimulation may be explained by difference in the ability of subjects to maintain attention to the stimulus. Attention to the stimulation and contraction may modulate the effect of the intervention on corticospinal excitability [51-54]. Despite instruction and reminders to focus on the stimulation every 5 min, attention may have been less in the 60-min protocol leading to a decreased response.

This study is the first to examine the effect of duration of motor ES (30 Hz, ramped) on corticospinal excitability. Our finding that 20-min of motor ES (30 Hz, ramped) is sufficient to produce a significant increase in corticospinal excitability that lasts at least 20-min supports the use of shorter stimulation periods in rehabilitation settings. Such duration of application is likely to be easier to administer and more efficient. The period of increased corticospinal excitability is likely to provide therapists with a window of opportunity to assist patients to learn novel tasks and aid skill acquisition [2,5,19-21]. However, further studies examining the link between increased corticospinal excitability, motor ES and learning are required before conclusions regarding potential clinical application can be made.

### Study limitations

This study has few limitations, however, two issues should be mentioned. One is the relatively short follow-up time of 20 min. Studies of 1–10 Hz motor ES report increased corticospinal excitability for up to 120 min after cessation of stimulation [26]. We are not aware of any evidence that temporal changes in corticospinal excitability depend on duration of motor ES. Further work should investigate this question. Second, this study used a relatively small sample size of 14 healthy subjects. Small sample sizes are common in TMS studies due to the novel and explorative aspect of TMS research. Caution must be exercised when interpreting these findings and extrapolating to the wider population. In addition, further testing of the duration of motor ES paradigms requires in-depth exploration on subjects with neurological pathology before clinical recommendations can be made.

## Conclusion

This study demonstrated that short durations of motor ES (20-min) are sufficient to increase corticospinal excitability in healthy subjects. Longer durations of motor ES do not appear to alter corticospinal excitability which may be due to homeostatic plasticity. The findings should be of interest to clinicians who aim to increase corticospinal excitability to assist with the rehabilitation of patients following neurological injuries that involve central nervous system lesions. However, further testing with larger sample sizes, measurement of the temporal effect of different duration applications and testing in neurological populations is required.

## Abbreviations

ANOVARM: Analysis of variance (repeated measures); APB: Abductor pollicis brevis; EMG: Electromyography; ES: Electrical stimulation; LTD: Long-term depression; LTP: Long-term potentiation; MEP: Motor evoked potential; Mmax: Maximal compound muscle action potential; TMS: Transcranial magnetic stimulation.

## Competing interests

The authors declare that they have no competing interests.

## Authors’ contributions

RA, SS and LC contributed to study design, data collection and analysis. All authors contributed to data interpretation, manuscript preparation and read and approved the final manuscript.
